# Chilaiditi’s Syndrome Mimicking Crohn’s

**DOI:** 10.7759/cureus.54655

**Published:** 2024-02-21

**Authors:** Sandres Aodish, Vincent Chang, Alexander Callow

**Affiliations:** 1 General Surgery, Lake Erie College of Osteopathic Medicine, Erie, USA; 2 General Surgery, Rochester Regional Health, Rochester, USA

**Keywords:** crohn's-mimicking, chilaiditi sign, crohn’s disease (cd), crohn’s-like disease, chilaiditi’s syndrome

## Abstract

Chilaiditi’s sign (colonic interposition) is a rare anomaly due to an abnormally located portion of the colon that is interposed in between the liver and the diaphragm. This rare anomaly is often incidentally seen on chest or abdominal radiographs. Chilaiditi’s radiographic sign is usually asymptomatic, whereas the medical condition accompanied by clinical symptoms is termed Chilaiditi’s syndrome. Possible causes of the syndrome include a long and mobile colon, scarring of the liver (cirrhosis), ascites, long-standing lung disease, as well as laxity of the falciform ligament. The most common clinical signs of Chilaiditi’s syndrome include gastrointestinal symptoms; however, clinical presentation can vary. This report describes a case of a 21-year-old male patient who presented with a longstanding history of left upper quadrant epigastric abdominal pain with diarrhea (six to eight loose watery stools). The patient was diagnosed with Crohn’s colitis and had tried a myriad of medical therapies with no adequate response. He chose to seek a second opinion and was subsequently discovered to have Chilaiditi’s syndrome via computed tomography (CT) and confirmed by barium enema. The patient then elected to undergo a right laparoscopic colectomy to resolve the symptoms. By postoperative day five, all symptoms had resolved including abdominal pain and diarrhea. Therefore, it is important to consider Chilaiditi’s syndrome as a differential diagnosis in persons presenting with left upper quadrant pain and symptoms of Crohn’s colitis, especially those treated with adequate medical therapy without alleviation of symptoms.

## Introduction

Chilaiditi’s sign (colonic interposition) is a rare anomaly that is incidentally seen on chest or abdominal radiographs, with an estimated incidence of 0.025-0.28% [1]. Chilaiditi’s radiographic sign is due to an abnormally located portion of the colon that is interposed in between the liver and the diaphragm. While the radiographic sign is usually asymptomatic, the medical condition accompanied by clinical symptoms is termed Chilaiditi’s syndrome. The most common clinical signs of Chilaiditi’s syndrome include gastrointestinal symptoms; however, clinical presentation can vary among persons. In rare cases, the condition is associated with breathing problems such as respiratory distress [2]. Chilaiditi syndrome equally affects males and females. The syndrome is more common in older adults but can occur at any age including children [3].

## Case presentation

A 21-year-old male presented with a longstanding history of left upper quadrant epigastric abdominal pain with diarrhea (six to eight loose watery stools). He was previously diagnosed with Crohn’s colitis at the age of 15 years old and was subsequently started on infliximab (Remicade) and then switched to adalimumab (Humira) due to decreased efficacy. The patient had also undergone a myriad of surgical procedures such as a cholecystectomy, appendectomy, and gastropexy for gastric volvulus. Despite medical and surgical interventions, the symptoms of left upper quadrant pain and diarrhea were not alleviated. Therefore, the patient chose to seek a second opinion.

**Figure 1:**
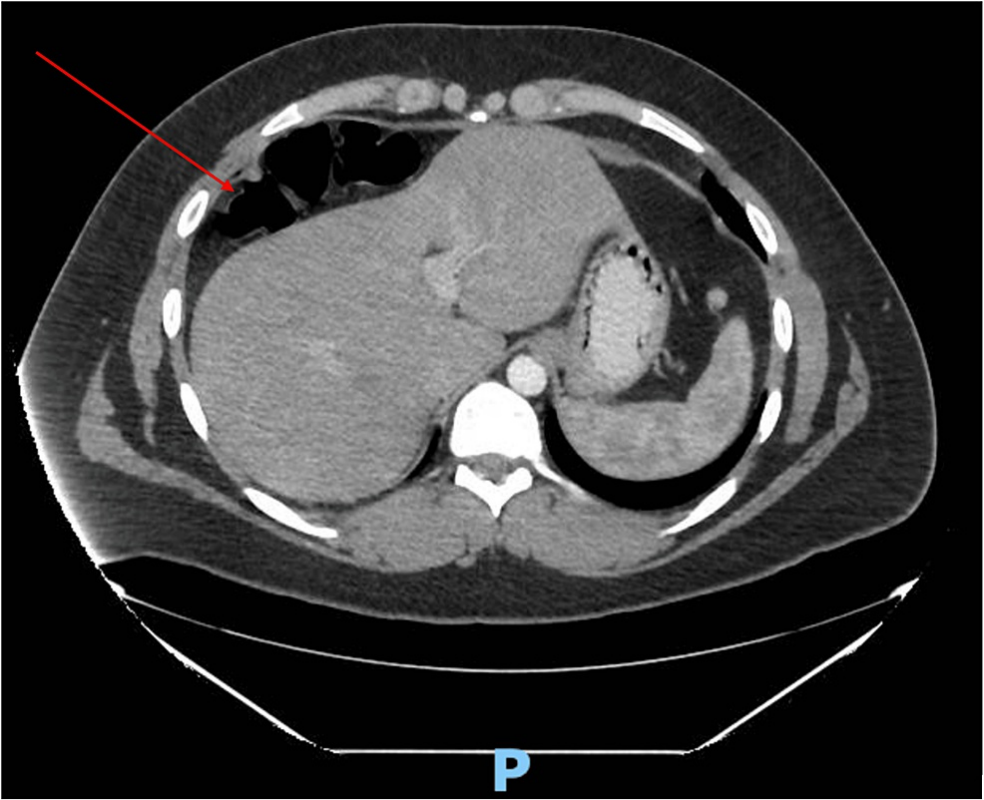
Axial CT of Right Colonic Interposition (Red Arrow)

**Figure 2:**
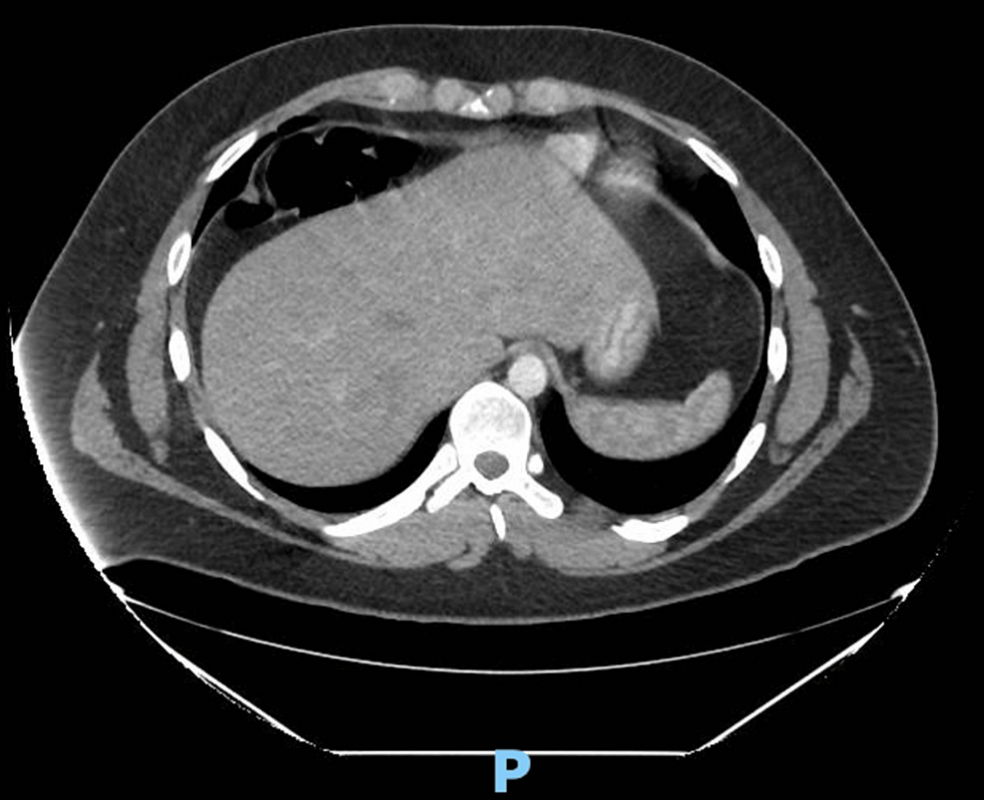
CT of Right Colonic Interposition

**Figure 3:**
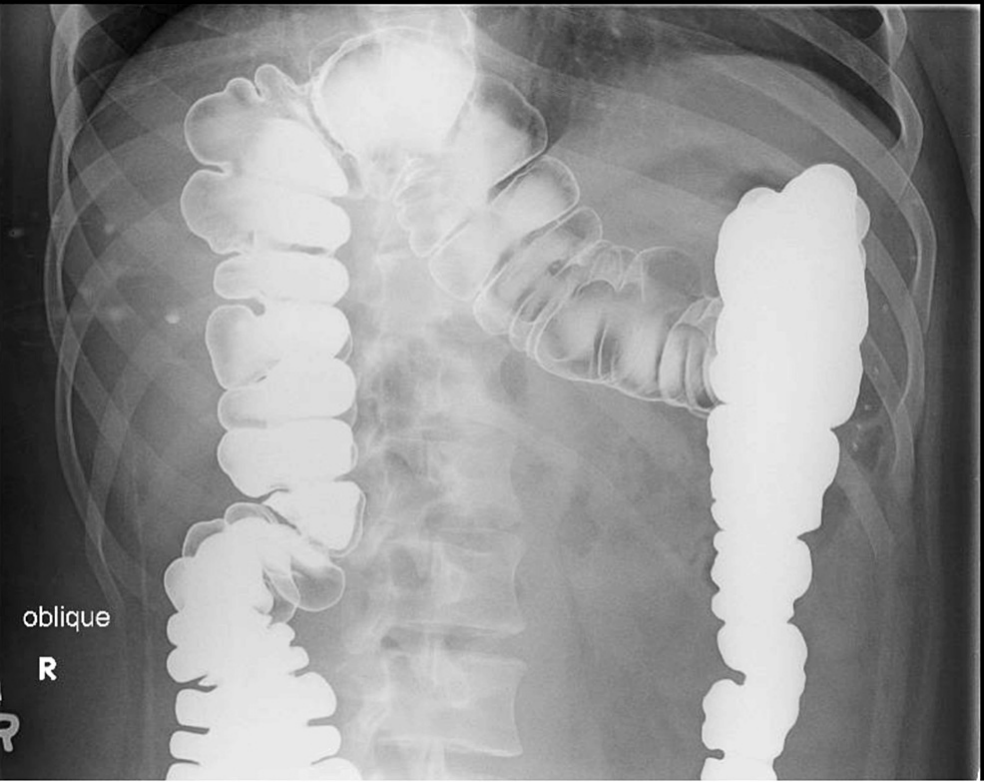
Barium Enema Confirming Chilaiditi's Syndrome

**Figure 4:**
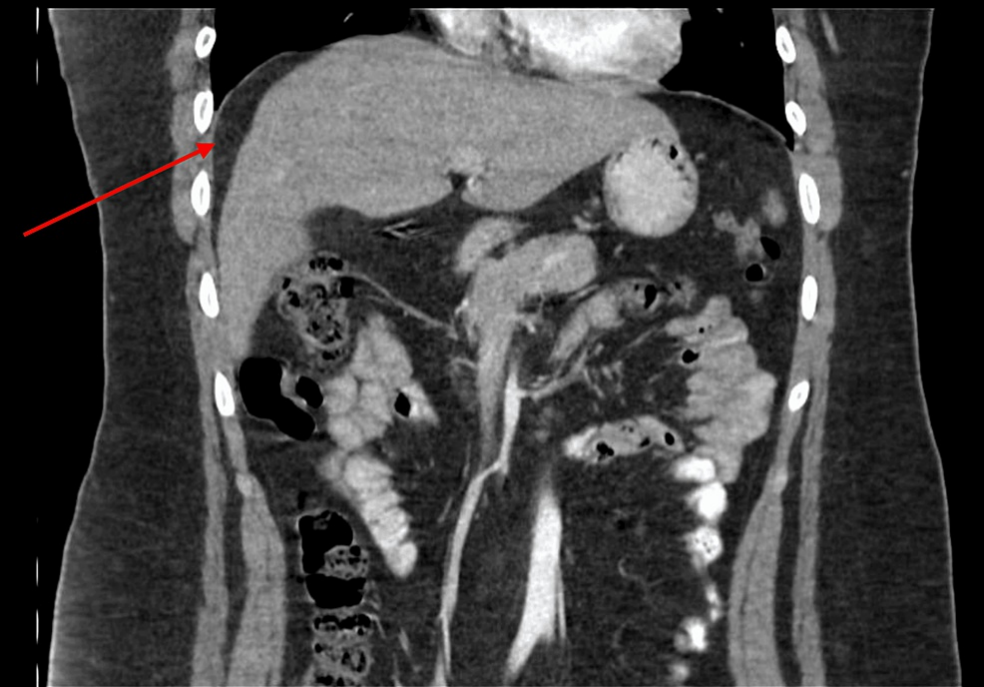
Coronal CT of Colonic Interposition (Red Arrow)

Computed tomography (CT) scan was suggestive of Chilaiditi’s syndrome as the right colon was lying superior anterior to the right lobe of the liver (Figures 1-3). The diagnosis was confirmed with a barium enema that showed a long redundant hepatic flexure of the colon lying above the liver consistent with Chilaiditi's syndrome (Figure 4). After being diagnosed with Chilaiditi’s syndrome, the patient initially decided not to undergo elective surgery. The patient continued to have episodic left upper epigastric quadrant pain which caused diarrhea and nausea, although he denied acute vomiting or intractable pain.

After seven months, the patient elected to undergo a right laparoscopic colectomy to resolve the symptoms. Intraoperatively, it was noted that the entire proximal transverse colon and hepatic flexure were lying on top of the liver with an attached omentum. Further inspection also revealed that the entire right colon was detached consistent with mobile cecal syndrome. The cecum, however, was normally positioned and noted not to be sinking deep into the pelvis. No hernias, volvulus, or adhesions were distinguished during the operation. The procedure progressed efficiently without any intraoperative complications. On postoperative day five, all symptoms had resolved including abdominal pain and diarrhea.

## Discussion

Literature review of PubMed/MEDLINE (Medical Literature Analysis and Retrieval System Online) and OstMed did not result in any previously published cases of Chilaiditi’s syndrome mimicking Crohn’s colitis. To our knowledge, this is the first case presentation of its kind. This rare anatomic anomaly was discovered in 1910 by a Greek radiologist named Dimitrios Chilaiditi. He discovered three cases in which patients had a right-sided hemi-diaphragmatic transposition of the colon. In modern times, this condition has been given the name Chilaiditi’s syndrome. This occurs when a portion of the large intestine slides superiorly between the liver and the diaphragm [4]. It should be noted that those without symptoms but with the transposition of the colon are said to have Chilaiditi’s sign; however, if symptoms develop it is then referred to as Chilaiditi syndrome. It has been suggested that there is a 4:1 male-to-female ratio incidence of Chilaiditi’s sign [5]. This rare condition can mimic other more common conditions such as respiratory compromise, angina-like symptoms, nausea, vomiting, diarrhea, bowel obstruction, pneumoperitoneum, and abdominal pain [5-7].

Due to the paucity and varying presentations of Chilaiditi's syndrome, it may be misinterpreted as a wide range of other conditions, which can subject patients to unnecessary treatments or invasive procedures. Economically, misinterpretation or failure to diagnose this syndrome can result in increased expenditures for not only the patient but the healthcare system as well. Physicians in the surgical and gastrointestinal subspecialties should be especially aware of Chilaiditi’s sign and syndrome. Inexperienced surgeons can easily misinterpret the radiographic sign as pneumoperitoneum resulting in further surgical exploration. The goal from a surgical perspective should be to evaluate if the subdiaphragmatic air is free or intraluminal before surgically exploring the abdomen to prevent needless complications [8]. Failure to do so can result in surgical complications such as infection and perforation. On the other hand, from a gastrointestinal perspective, a patient may be subjected to a possibly needless colonoscopy such as in this case. The colonoscopy could have potentially resulted in perforation caused by the increased risk of air entrapment in the interposed bowel [8].

Treatment of Chilaiditi’s syndrome is usually conservative and is directed towards specific symptoms for each individual [3]. Misdiagnosis or failure to include Chilaiditi’s sign or syndrome as a differential can result in the administration of medications with high adverse effect profiles or invasive procedures without treating the underlying issue. This is in direct opposition to an initial low-cost conservative approach used for Chilaiditi’s syndrome such as bed rest, intravenous fluids, enemas, and laxatives [1]. Expensive surgical interventions requiring the removal of a portion of the colon such as transverse colectomy or right-sided colectomy are only performed once conservative methods do not alleviate symptoms.

The economic burden on the patient from various medication trials and unsuccessful surgical interventions can be financially detrimental. With the ever-rising cost of healthcare, hospital systems and organizations can be negatively impacted financially as well. Expenditures incurred by the healthcare system include frequent hospital readmissions, increased staff utilization, unnecessary medication use, and prolonged length of stay resulting in decreased reimbursements. Thus, it is crucial to accurately recognize Chilaiditi’s sign or syndrome to prevent unnecessary medical interventions and costs. This case specifically serves as an example to consider Chilaiditi’s syndrome as a differential in patients with refractory Crohn’s colitis but should also be considered as part of a differential diagnosis in other common abdominal conditions.

## Conclusions

We presented a rare case of a young male diagnosed with Crohn’s colitis during adolescence who presented with left upper quadrant abdominal pain and watery diarrhea. The patient was medically treated for Crohn’s colitis without improvement of symptoms. Radiographic CT studies showed colonic interposition suggestive of Chilaiditi's syndrome with confirmatory diagnosis via barium enema. After electing to undergo a right laparoscopic colectomy, the patient had a resolution of symptoms within five days. Therefore, it is important to consider Chilaiditi’s syndrome as a differential diagnosis in persons presenting with left upper quadrant pain and symptoms of Crohn’s colitis.
